# Synergetic effect in water treatment with mesoporous TiO_2_/BDD hybrid electrode†

**DOI:** 10.1039/c9ra10318j

**Published:** 2020-01-08

**Authors:** Norihiro Suzuki, Akihiro Okazaki, Haruo Kuriyama, Izumi Serizawa, Yuki Hirami, Aiga Hara, Yuiri Hirano, Yukihiro Nakabayashi, Nitish Roy, Chiaki Terashima, Kazuya Nakata, Ken-ichi Katsumata, Takeshi Kondo, Makoto Yuasa, Akira Fujishima

**Affiliations:** Photocatalysis International Research Center, Research Institute for Science and Technology, Tokyo University of Science 2641 Yamazaki Noda Chiba 278-8510 Japan suzuki.norihiro@rs.tsu.ac.jp; ORC Manufacturing Co., Ltd 4896 Tamagawa Chino Nagano 391-0011 Japan; Faculty of Science and Technology, Tokyo University of Science 2641 Yamazaki Noda Chiba 278-8510 Japan; Faculty of Industrial Science and Technology, Tokyo University of Science 6-3-1 Niijyuku, Katsushika Tokyo 125-8585 Japan; Faculty and Graduate School of Urban Environmental Sciences, Department of Applied Chemistry for Environment, Tokyo Metropolitan University 1-1 Minami-Osawa Hachioji Tokyo 192-0397 Japan; Department of Chemistry, University of North Bengal Raja Rammohunpur Darjeeling West Bengal-734013 India; Graduate School of Bio-Applications and Systems Engineering, Tokyo University of Agriculture and Technology 2-24-16 Nakacho Koganei Tokyo 184-0012 Japan

## Abstract

Boron-doped diamond (BDD) electrodes have a wide potential window and can produce ozone by water electrolysis at high voltage. Though ozone has strong oxidative power (standard oxidation potential: 2.07 V *vs.* NHE), it cannot decompose certain types of recalcitrant organic matter completely. We developed an advanced oxidation process (AOP), in which hydroxy radicals with stronger oxidative power (standard oxidation potential: 2.85 V *vs.* NHE) are formed using a combination of ozone, photocatalyst, and UV. In this study, we fabricated a mesoporous TiO_2_/BDD hybrid electrode and examined its potential for AOPs. A synergetic effect between electrochemical water treatment and photocatalytic water treatment was observed with the hybrid electrode that did not occur with the BDD electrode.

## Abstract 2

A mesoporous TiO_2_/BDD hybrid electrode showed a synergetic effect between electrochemical water treatment and photocatalytic water treatment.

## Graphical abstract

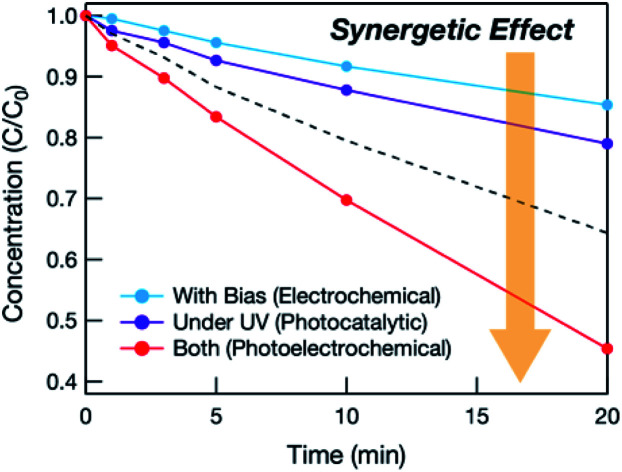

## 1. Introduction

Boron-doped diamond (BDD) electrodes have a wide potential window, and low background current, and physical/chemical stability.^1^ The wide potential window allows BDD to be used for the production of ozone (O_3_) by the electrolysis of water at high voltage. Because ozone is a strong oxidizing agent (standard oxidation potential: 2.07 V *vs.* Normal Hydrogen Electrode (NHE))^2^ that decomposes to form harmless oxygen (O_2_), ozonation has attracted great interest as an environmentally benign water purification technique. However, the oxidative power of O_3_ is sometimes insufficient to decompose recalcitrant organic compounds that are widely used as agricultural chemicals, medicines, dyes, and industrial products.

Advanced oxidation processes (AOPs), in which UV light, photocatalysts, and O_3_ are used together, have been studied as methods to more effectively treat contaminated water. These processes aim to efficiently produce hydroxy radicals (OH˙), which have greater oxidative power (standard oxidation potential: 2.80 V *vs.* NHE)^2^ than oxidants such as O_3_ and H_2_O_2_ (standard oxidation potential: 1.77 V *vs.* NHE).^2^ Ochiai *et al.* proposed an AOP system in which O_3_ gas was introduced into a reactor containing a titanium mesh impregnated photocatalyst (TMiP) and an ultraviolet light source.^3^ Their study found that using the photocatalyst and O_3_ in combination resulted in greater degradation of phenol in water than using either the photocatalyst or ozone individually. Water treatment using O_3_ produced by a water electrolysis unit composed of a BDD anode and a Pt cathode was found to deactivate water-borne pathogens.^4^ This water electrolysis unit used in combination with a photocatalyst was also found to effectively decompose toxic chemicals (perfluorooctanoic acid, C_7_F_15_COOH).^5^ The system used in this study was large because the electrochemical and photocatalytic units were separate, but simpler and more compact AOP systems are preferable for a wide variety of uses.

A BDD photocatalyst combined with water electrolysis to produce O_3_ under illumination results in a simpler AOP system because the photocatalytic and electrochemical treatment of water treatment can be performed simultaneously. Previous studies of nanostructured composite n-type metal oxides (TiO_2_, ZnO) and BDD found that their photocatalytic performance was enhanced because of photocarrier separation at the hetero-interface.^6^ However, no voltage was applied to these composites, and electrochemical effects were not studied. Recently, Zhao *et al.* synthesized a TiO_2_/Sb-doped SnO_2_/BDD hybrid electrode with a double-layer three-dimensional macro–mesoporous structure. This hybrid electrode showed superior water treatment performance when it was used as a photoanode because of the accumulation of photogenerated holes on the surface under a strong positive potential.^7^ However, the voltage applied to this hybrid electrode was not high enough to produce O_3_ by water electrolysis. Therefore, it seems that the potential of this hybrid electrode has not been fully explored.

In our previous study, we fabricated a mesoporous TiO_2_ layer on BDD and found that its photocatalytic efficiency was enhanced by deep-UV illumination.^8^ In this paper, we used this hybrid electrode to electrolyze water to produce O_3_ and other reactive oxygen species (ROS). In addition, we conducted water treatment tests with the electrochemically-created ROS under UV light irradiation and demonstrated the capability of the system for an AOP.

## 2. Experimental

### 2.1 Materials

Phosphate buffer powder (consisting of Na_2_HPO_4_ and KH_2_PO_4_), methylene blue, sodium hydroxide (NaOH), potassium iodide (KI), and ammonium molybdate tetrahydrate ((NH_4_)_6_Mo_7_O_24_·4H_2_O) were obtained from FUJIFILM Wako Pure Chemicals Corporation (Osaka, Japan). Potassium hydrogen phthalate (C_6_H_4_(COOK)(COOH)) was purchased from Tokyo Chemical Industry Co, Ltd (Tokyo, Japan). These chemicals were used without further purification.

### 2.2 Electrochemical analysis for mesoporous TiO_2_/BDD hybrid electrode

Mesoporous TiO_2_/BDD hybrid electrodes were synthesized as described in our previous study.^8^ Cyclic voltammograms were measured using a VersaSTAT 4 (AMETEK, Berwyn, PA, USA), while the relationship between applied voltage and current was examined using a HABF5001-B10 potentiostat/galvanostat (HOKUTO DENKO, Tokyo, Japan). A BDD electrode (without the TiO_2_ layer) was used as a reference. A 0.25 M phosphate aqueous buffer solution (pH = 6.8) was used as the electrolyte.

### 2.3 Detection of reactive oxygen species (ROS)

To prevent reduction by hydrogen gas resulting from the electrolysis of water, an H-type cell consisting of two separate glass half-cells was used and the mesoporous TiO_2_/BDD hybrid electrode (working electrode) and Pt (counter electrode) were placed in different half-cells. The half-cells were connected by a Nafion® NRE-212 membrane (Sigma-Aldrich, St. Louis, MO, USA), and 50 mL of 0.25 M phosphate aqueous buffer solution (pH = 6.8) was added to each one to increase the conductivity of the water in the half-cells and maintain its pH during the experiment.

Ozone gas was detected using a Kitagawa gas detector tube system consisting of an air sampling pump and a precision gas detector tube. Prior to the measurement, the 0.25 M phosphate aqueous buffer solution in the half-cells with BDD or hybrid electrodes was purged with N_2_ gas for 30 min to remove dissolved oxygen. A constant current (50 mA, 75 mA, or 100 mA) was then applied to the electrodes (hybrid electrode or BDD) for 3 min while N_2_ gas was bubbled through the solution at a constant flow rate of 0.15 L min^−1^. The gas flow rate was maintained using an RK-1650 flow meter (KOFLOC, Kyotanabe, Japan). The outlet gas was collected in 2L Tedlar® bags and passed through a 182U O_3_ detector tube (KOMYO RIKAGAKU KOGYO K.K., Kawasaki, Japan) to determine how much O_3_ gas was produced. A schematic illustration of this experimental setup is shown in Fig. S1(a).†

The quantity of H_2_O_2_ formed by the application of current to the electrodes (either hybrid or BDD) for 10 min was determined using the coloring reaction.^9^ To detect H_2_O_2_, 500 mL of an aqueous solution of 1 g of NaOH, 33 g of KI, and 0.1 g of (NH_4_)_6_Mo_7_O_24_·4H_2_O (Solution A) and 500 mL of an aqueous solution of 10 g of C_6_H_4_(COOK)(COOH) (Solution B) were first separately prepared. Treated water, Solution A, and Solution B were then mixed in a 1 : 1 : 1 ratio. If H_2_O_2_ exists in the treated water, I_3_^−^ (which has an absorption maximum at approximately 350 nm) is formed by the following reactions:

1 H_2_O_2_ + 2I^−^ + 2H^+^ → I_2_ + 2H_2_O

2 I_2_ + I^−^ ⇄ I_3_^−^

The concentration of H_2_O_2_ was estimated from the peak intensity (at around 350 nm) of the optical absorption spectrum measured by a V-670 UV-VIS spectrometer (JASCO, Hachioji, Japan).

### 2.4 Water treatment test

The efficacy of water treatment was evaluated based on the decomposition of methylene blue (MB). The glass half-cell containing the mesoporous TiO_2_/BDD hybrid electrode was filled with 50 mL of a solution of MB (20 μM) dissolved in 0.25 M phosphate aqueous buffer solution, while the other half-cell (*i.e.*, the Pt electrode side) was filled with the same amount of pure phosphate aqueous buffer solution (0.25 M). The hybrid electrode was placed horizontally in the dark until adsorption equilibrium was reached. It was then irradiated with 1.2 mW cm^−2^ of 222 nm UV light from an excimer lamp (ORC Manufacturing Co., Ltd, Machida, Japan) in a quartz sample tube. A schematic illustration is shown in Fig. S1(b).† The glass cup was capped with a Teflon cap to prevent evaporation during the test, and samples of the MB solution were taken by opening the cap. The absorption spectrum of the MB solution was measured using a V-670 UV-VIS spectrometer (JASCO, Hachioji, Japan). During the test, the MB solution was constantly stirred.

## 3. Results and discussion

**Fig. 1:**
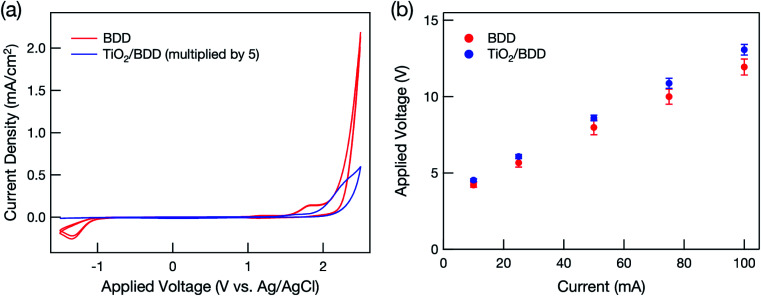
(a) Typical cyclic voltammogram of BDD and mesoporous TiO_2_/BDD hybrid electrodes in a 0.25 M phosphate aqueous buffer solution at a scan rate of 0.1 V s^−1^. The current density in the hybrid electrode was multiplied by five. (b) Relationship between applied voltage and current in BDD and mesoporous TiO_2_/BDD hybrid electrodes in a 0.25 M phosphate aqueous buffer solution.

Fig. 1(a) summarizes the cyclic voltammograms. Wide potential windows, which are typical of BDD electrodes, were clearly observed and were retained even after modification with mesoporous TiO_2_. In the hybrid electrode, current density decreased dramatically because most of the BDD surface was covered with TiO_2_, although some BDD remained exposed through mesopores and/or cracks (Fig. S2†).

Water oxidation typically produces O_2_ at the anode by the following reaction:

3 2H_2_O → O_2_ + 4H^+^ + 4e^−^

When BDD is used as an anode, the voltage required to electrolyze water becomes higher than the theoretical value (1.23 V) due to its wide potential window. This leads to other reactions such as O_3_ production by water electrolysis:

4 3H_2_O → O_3_ + 6H^+^ + 6e^−^

Fig. 1(b) shows that at high voltage, the current flow increased linearly with applied voltage in both electrodes and that the mesoporous TiO_2_ layer had little influence on the current flow.

**Fig. 2:**
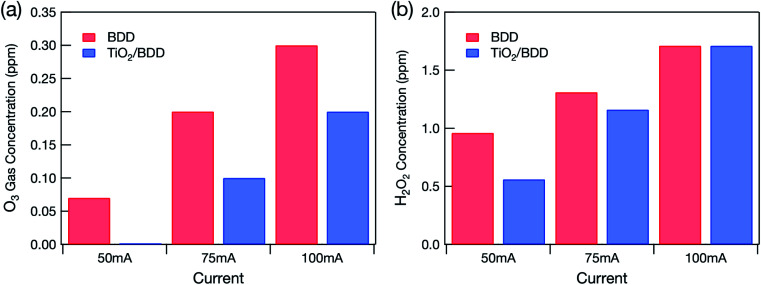
Concentrations of (a) O_3_ gas from the glass cell and (b) H_2_O_2_ dissolved in treated water caused by the application of different constant currents to BDD and mesoporous TiO_2_/BDD hybrid electrodes.

Fig. 2 shows the quantities of reactive oxygen species detected. The quantity of O_3_ gas produced by the electrolysis of water (eqn (4)) increased linearly with the applied current (Fig. 2(a)). The mesoporous TiO_2_/BDD hybrid electrode produced less O_3_ gas than the BDD electrode because it had less exposed BDD. Indeed, no O_3_ gas was detected when a current of 50 mA was applied to the hybrid electrode.

If O_3_ is dissolved in the treated water, an absorption peak at around 260 nm occurs in the UV-VIS spectrum.^10^ However, no such peak was detected in the treated water (not shown). Hydrogen peroxide (H_2_O_2_) was, however, detected in the treated water in quantities that increased linearly with the current applied to the electrodes (Fig. 2(b)). Because high voltage was applied to electrodes, not only O_3_ but also other highly oxidative species (H_2_O_2_ and OH˙) were produced by the electrolysis of water:

5 2H_2_O → H_2_O_2_ + 2H^+^ + 2e^−^

6 H_2_O → OH˙ + H^+^ + e^−^

Therefore, substantial direct production of H_2_O_2_ by water electrolysis (eqn (5)) and annihilation of the OH˙ produced by water electrolysis (eqn (7)) were observed.

7 2OH˙ → H_2_O_2_

However, O_3_ gas production (eqn (4)) predominates at lower bias voltage. Most of the H_2_O_2_ was thus produced by the reaction between O_3_ gas and water. A previous study revealed that dissolved O_3_ forms H_2_O_2_ by the following chain reactions:^11^8
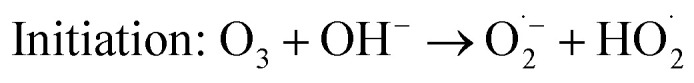
9
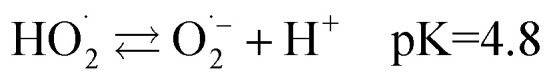
10
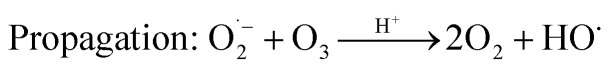

11 HO˙ + O_3_ → {O_3_OH} → HO^˙^_2_ + O_2_

12 Termination: 2{O_3_OH} → H_2_O_2_ + 2O_3_

Because the hybrid electrode produced less O_3_ gas, the H_2_O_2_ concentration in the treated water was also lower. However, when a current of 100 mA was applied, the H_2_O_2_ concentration in the treated water became almost the same as when the BDD electrode was used. This probably occurred because the TiO_2_ layer peeled off.

We decided to apply a 75 mA current to the electrodes for further studies by following reasons. Lower current (50 mA) will lead to the formation of OH˙ in BDD. However, because O_3_ gas was not detected from the hybrid electrode, while it was formed from BDD, an experimental condition becomes different. At higher current (100 mA), if TiO_2_ layer in the hybrid electrode was truly peeled off, there are no difference between the hybrid electrode we made and the BDD electrode. Even if not, once BDD electrode is at a relatively high potential, the OH˙ will easily to take further reaction to form other oxidative species, which reduces the water treatment ability in BDD. Therefore, a comparative experiment between these two electrode is difficult at lower and higher current.

**Fig. 3:**
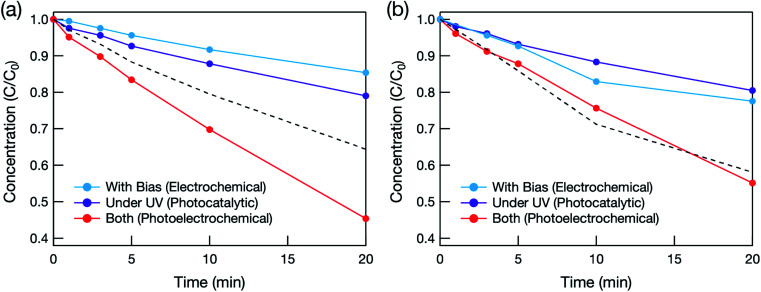
Time dependence of MB concentration estimated from the visible spectra during the photocatalytic (under 222 nm illumination without bias voltage), electrochemical (without illumination while bias voltage was applied), and photoelectrochemical (under 222 nm illumination with bias voltage) water treatment tests of (a) mesoporous TiO_2_/BDD hybrid and (b) BDD electrodes. *C* and *C*_0_ are the remaining and initial concentrations of MB, respectively. The black dotted line is a simulated value assuming that the total amount of decomposed MB is a summation of the amounts of MB decomposed by the photocatalytic and electrochemical effects.

The effectiveness of the hybrid electrode for water treatment was evaluated based on the decomposition of methylene blue (MB). Photocatalytic water treatment was conducted under deep-UV light (*λ* = 222 nm) in order to photoexcite not only TiO_2_ but also BDD, which has a band gap of 5.5 eV (*λ* = 225 nm). Fig. 3 shows the relationship between MB concentration and photoirradiation time. When the hybrid electrode was used with deep-UV irradiation (Fig. 3(a)), the total amount of MB decomposed was greater than the sum of the amounts decomposed photocatalytically and electrochemically. This synergetic effect was not observed when the BDD electrode was used (Fig. 3(b)). The synergetic effect therefore originated from the mesoporous TiO_2_ layer. Deep-UV light can form OH˙ by decomposing O_3_ and H_2_O_2_ by the following reactions:^12^

13 O_3_ + *hν* → O^1^(D) + O_2_

14 O^1^(D) + H_2_O → H_2_O_2_

15 H_2_O_2_ + *hν* → 2HO˙

However, the synergetic effect did not occur in the pure BDD electrode (without the mesoporous TiO_2_ layer), probably because the light intensity was too weak (1.2 mW cm^−2^).

Photoexcitation in TiO_2_ creates holes (h_vb_^+^) in the valence band (VB) that directly oxidize MB and/or form OH˙ *via* surface oxygen ions of the TiO_2_ lattice (O_s_^2−^):^13^

16 O_s_^2−^ + H_aq_^+^ + h_vb_^+^ → OH˙

By contrast, electrons (e_cb_^−^) in the conduction band (CB) reduce the dissolved O_2_ to form superoxide radicals 
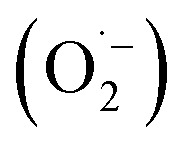
:17
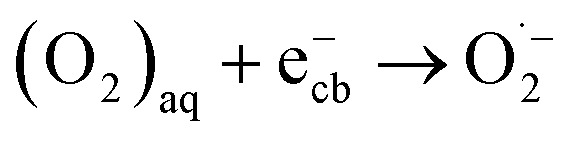


from which OH˙ can then be formed by the following sequence of reactions.^13^18
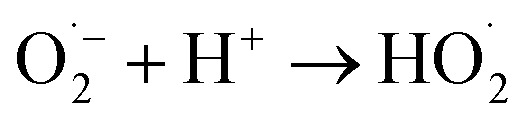

19 HO_2_˙ + H^+^ + e_cb_^−^ → H_2_O_2_

20 Ti^3+^ + H_2_O_2_ → Ti^4+^ + OH˙ + OH^−^

21
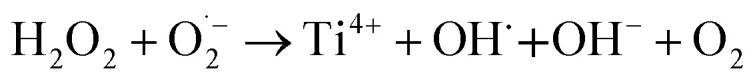


**Fig. 4:**
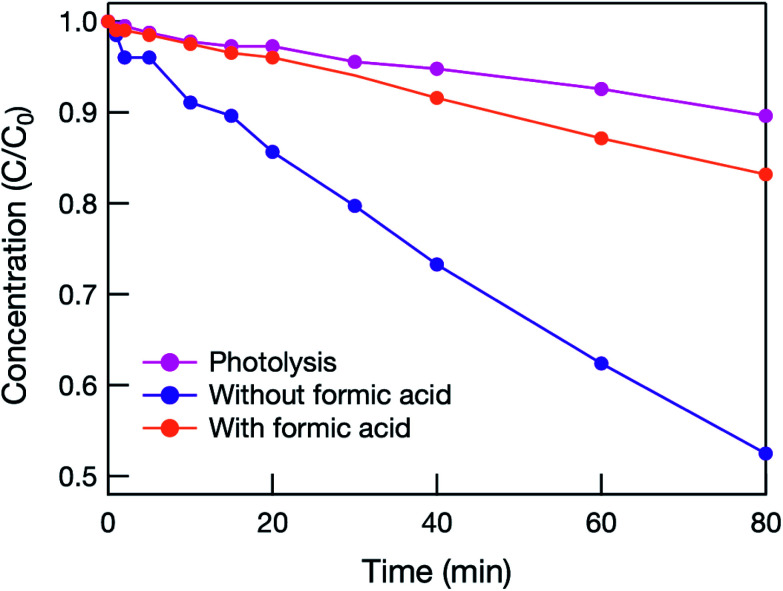
Time dependence of MB concentration estimated from visible spectra during the photocatalytic (under 222 nm illumination) water treatment test of mesoporous TiO_2_/BDD hybrid electrode with and without 1 mM formic acid (a sacrificial reagent added to react with holes). *C* and *C*_0_ are the remaining and initial concentrations of MB, respectively.

To clarify the mechanism of the photocatalytic reaction, formic acid was added to react sacrificially with holes.^7^ As shown in Fig. 4, when formic acid was added, the photocatalytic activity decreased dramatically, and the initial MB concentration change was almost identical to that caused by photolysis alone. This means that oxidation induced by holes in the VB was the main factor, and electrons in the CB were not used sufficiently to decompose MB in the hybrid electrode. This is because the oxidative power of 
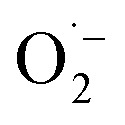
 is much lower than those of h_vb_^+^ and OH˙.^14^

Under electrochemical conditions (*i.e.*, with current applied to the electrode), as mentioned above, H_2_O_2_ was produced in the treated water. This is probably because the reduction potential of the H_2_O_2_/OH˙ couple is more positive (*E* = +0.87 V *vs.* NHE)^15^ than that of the 
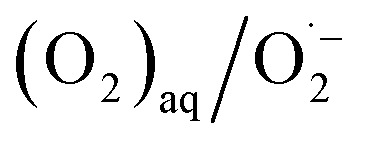
 couple (*E* = −0.16 V *vs.* NHE; at a concentration of 1 M in water),^16^ photoexcited electrons (e_cb_^−^) in the CV of TiO_2_ preferentially reduced electrochemically formed H_2_O_2_ to form OH˙.^17^

22 H_2_O_2_ + e_cb_^−^ → OH˙ + OH^−^

Electrons in the conduction band therefore played a significant role in decomposing MB by creating more OH˙ and thus promoting the advanced oxidation process. Such H_2_O_2_-assisted photocatalytic reactions have been reported previously.^17^

## 4. Conclusions

Mesoporous TiO_2_/BDD hybrid electrodes were prepared using a surfactant-assisted sol–gel method. By applying a current, O_3_ gas was produced by the electrolysis of water, and dissolved O_3_ gas formed H_2_O_2_ through a chain reaction. Under UV irradiation, electrochemically-formed H_2_O_2_ was reduced by photoexcited electrons in the CB of TiO_2_ to form OH˙, which promotes MB decomposition because of its strong oxidative power. A synergetic effect occurred, demonstrating that the hybrid electrode is effective in promoting AOPs. This simple water treatment system can be applied to decompose recalcitrant organic compounds in wastewater, and further studies of this process are currently underway.

## Author Contributions

A. O., A. H., Y. H., Y. N., and N. R. synthesized the BDD electrode. N. S. and A. O. fabricated the mesoporous TiO_2_ thin film on the BDD electrode and conducted the experiments. Y. H. and C. T. gave scientific suggestions on this work. H. K. and I. S. supported this work financially. K. N., K.-i. K., T. K., and M. Y. contributed to the arrangement of the research environment. A. F. acted as a supervisor.

## Conflicts of interest

There are no conflicts to declare.

## Supplementary Material

RA-010-C9RA10318J-s001
