# Supplementary material for: Synergetic effect in water treatment with mesoporous TiO2/BDD hybrid electrode
Source: RSC Adv. 2020 Jan 8;10(3):1793–8. doi: 10.1039/c9ra10318j (PMC9047568; doi:10.1039/c9ra10318j)
Supplement: RA-010-C9RA10318J-s001 [file RA-010-C9RA10318J-s001.pdf]

Electronic Supplementary Material (ESI) for RSC Advances.
This journal is © The Royal Society of Chemistry 2020

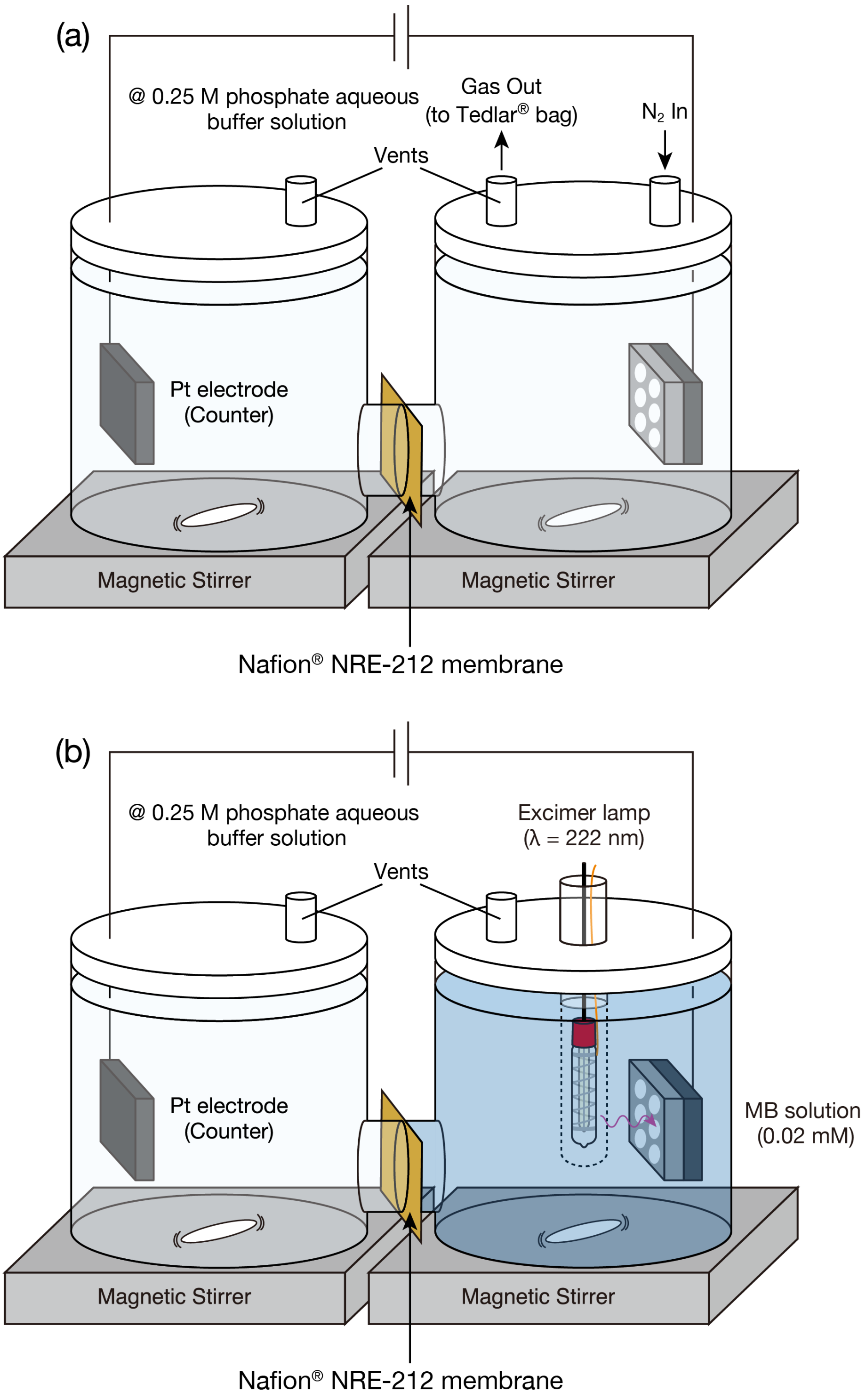

Figure S1. Schematic illustration of experimental setups for (a) detection of ozone gas produced during water electrolysis and (b) water treatment test using MB.

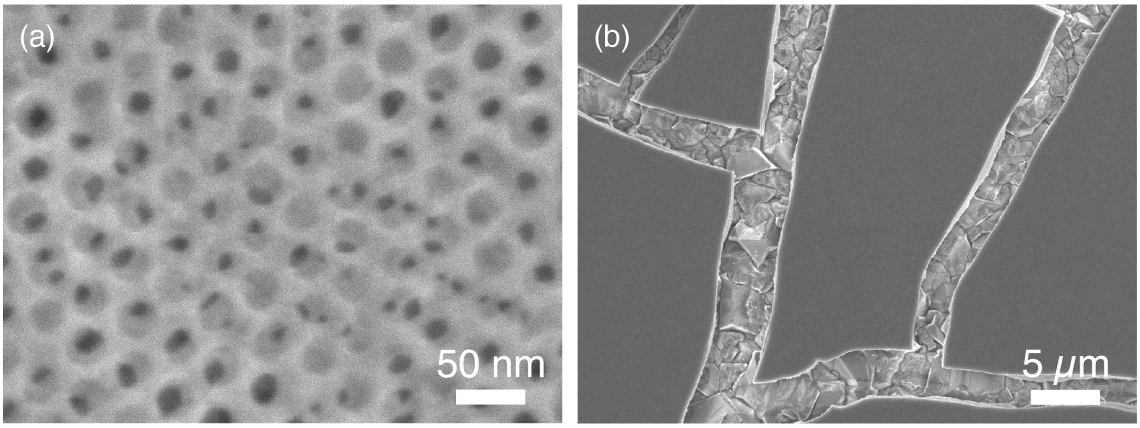

Figure S2. (a) Top view and (b) low-magnification SEM images of hybrid electrode. This figure has been reproduced from N. Suzuki *et al.*[8] in accordance with the Creative Commons Attribution (CC BY) license.
